# Application of Rapid Evaporative Ionization Mass Spectrometry (REIMS) to Identify Antimicrobial Resistance in Uropathogenic *Escherichia coli* (UPEC) Isolates via Deuterium Isotope Probing

**DOI:** 10.1021/acs.analchem.5c00667

**Published:** 2025-08-22

**Authors:** Sahand Shams, Sara Sadia Chowdhury, Joel Doherty, Shwan Ahmed, Dakshat Trivedi, Yun Xu, Joscelyn Sarsby, Claire E Eyers, Adam Burke, Royston Goodacre, Howbeer Muhamadali

**Affiliations:** 1 Centre for Metabolomics Research, Department of Biochemistry, Cell and Systems Biology, Institute of Systems, Molecular and Integrative Biology, 4591 University of Liverpool, Liverpool L69 7ZB, United Kingdom; 2 Centre for Proteome Research, Department of Biochemistry, Cell and Systems Biology, Institute of Systems, Molecular and Integrative Biology, 4591 University of Liverpool, Liverpool L69 7ZB, United Kingdom

## Abstract

Antimicrobial resistance (AMR) continues to pose a significant threat to global health, undermining advances in modern medicine and increasing mortality from previously treatable infections. Rapid and accurate antimicrobial susceptibility testing (AST) is critical, both for effective judicious treatment and controlling the spread of AMR. For the first time, we demonstrate the application of rapid evaporative ionization mass spectrometry (REIMS), combined with deuterium isotope probing (DIP), as a novel approach for identifying AMR in uropathogenic *Escherichia coli* (UPEC) isolates within only a 1 h incubation period. By directly analyzing bacterial samples without extensive preparation, REIMS serves as a rapid fingerprinting tool, employing DIP and multivariate statistical analysis to provide AST profiling of UPEC isolates. Distinct clustering patterns were observed between trimethoprim-susceptible and trimethoprim-resistant UPEC isolates grown in media containing 10% deuterium oxide (D_2_O). TMP-susceptible isolates treated with trimethoprim displayed no significant deuterium incorporation, serving as an indicator of a lower metabolic activity resulting from antimicrobial action. We also demonstrated the ability to differentiate the origin of heavy water, confirming that deuterium incorporation was a biological process rather than of extracellular origin resulting from chemical processes. Several mass spectral bins showed patterns consistent with deuterated phospholipid species, including those in the expected mass range for phosphatidylethanolamine (PE) and phosphatidylglycerol (PG), which are the most abundant phospholipids in *E. coli*. However, these annotations remain tentative, as no structural confirmation (e.g., MS/MS) was performed. These findings suggest that REIMS, combined with DIP and multivariate statistical analysis, serves as an efficient fast workflow for the rapid detection of AMR.

## Graphical abstract

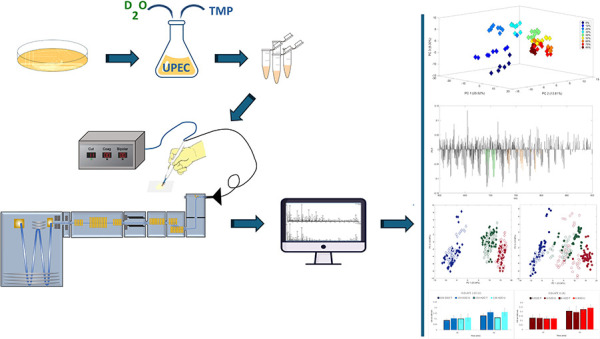

## Introduction

The advent of antibiotics has undeniably transformed medical practice, facilitating advancements in various fields such as surgical interventions, safer childbirth, and organ transplantation. Despite these significant achievements, the increase in antimicrobial resistance (AMR) poses a substantial threat to this progress. AMR is defined as a characteristic of any infectious pathogen that resists one or more antimicrobials previously effective in treating the infection.^1^ The global impact of AMR is challenging to quantify due to the lack of comprehensive epidemiological data in many regions. Nonetheless, the available data indicates a significant and growing concern. Projections estimate that if effective measures are not implemented, AMR could result in over 10 million deaths annually by 2050, surpassing the annual death tolls of diabetes and cancer combined, which currently stand at 1.5 million and 8.2 million, respectively.^2^ This alarming trend underscores the need for urgent global action to address AMR and preserve the progress made in modern medicine.^3^ Rapid detection of AMR pathogens ensures effective targeted treatment, thereby reducing unnecessary use of broad-spectrum antibiotics, and lowering the potential development and spread of AMR.^4^ AMR detection and antimicrobial susceptibility testing (AST) are essential for addressing the current AMR crisis. While traditional AST methods are reliable, they are time-consuming, labor-intensive, and prone to human error.^5^ Advances such as polymerase chain reaction (PCR) and nucleic acid amplification tests (NAATs) have enabled rapid and precise AMR identification, when the AMR genetic loci are known, while whole genome sequencing (WGS) provides comprehensive insights into resistance mechanisms.^6^ However, when compared to traditional phenotypic tests, these approaches are both costly and technically demanding and prior-establishment of AMR loci does not mean that it is expressed.

Metabolomics, the comprehensive study of metabolites in biological samples, provides powerful tools for AMR detection. These techniques including metabolite profiling, metabolic fingerprinting and footprinting, which enable rapid minimally invasive analysis of bacterial samples.^7,8^ Metabolic fingerprinting, in particular, can rapidly classify samples based on their biological characteristics, often in less than a minute without the need for chromatographic separation.^9^ Over the past decade, spectroscopy-based metabolic fingerprinting techniques, such as Raman, Fourier transform infrared (FT-IR), and optical photothermal infrared (O-PTIR) spectroscopy, have been employed to classify microorganisms accurately and identify AMR.^10–13^ These methods provide detailed molecular insights but typically achieve less than 90% accuracy.^14–16^ In contrast, mass spectrometry-based techniques, such as matrix-assisted laser desorption/ionization-time-of-flight-mass spectrometry (MALDI-TOF-MS), offer very high accuracy (exceeding 95%) for bacterial identification and AMR detection.^17–19^ MALDI-TOF-MS has significantly advanced bacterial identification and AMR detection in clinical settings. However, extensive processing and optimization for different matrixes are required in order to achieve reliable signal/noise data, prolonging turnover times. Furthermore, current procedures are unable to identify critical clinical phenotypes such as virulence and serotype(s), necessitating additional conventional testing. Technical limitations such as spectral interference, particularly with *Clostridium* species, further underscore the need for enhancements in sample preparation, functional classification, and workflow efficiency.^20,21^

To address these challenges, recent advancements in ambient ionization mass spectrometry, such as rapid evaporative ionization mass spectrometry (REIMS), have emerged as promising alternatives.^22^ These techniques enable the direct analysis of biological samples under atmospheric conditions, eliminating the need for preparative steps. By directly obtaining mass spectral fingerprints from intact bacterial cells, REIMS facilitates real-time, direct analysis.^23^ Although earlier mass spectrometry techniques including FAB-MS,^24^ ESI-MS,^25^ MALDI-MS,^26^ and DESI-MS,^27^ could profile bacterial lipids, however, they often face challenges in standardizing identification systems for bacterial species using intact phospholipids. One key advantage of REIMS is that culture media do not impact species classification,^21,28^ allowing bacteria to be grown in their optimal conditions without affecting the results. Additionally, REIMS enables direct analysis without the need for sample extraction, making it a promising approach for rapid and efficient bacterial profiling.^23,29^ The application of a radiofrequency electrical current to microbial biomass generates gas-phase ions from intracellular and extracellular metabolites, which are then subjected to immediate chemical analysis. This approach allows for species-level identification of bacterial isolates in just 2–3 s, offering a rapid, preparation-free solution that has the potential to transform current diagnostic workflows.^28^

Stable isotope probing (SIP) further enhances the capacity to monitor bacterial metabolic activity alongside AMR detection.^13,30–33^ SIP uses stable isotopes (e.g., ^2^H, ^13^C, ^15^N, ^18^O) as tracers in biochemical systems, enabling detection via techniques like mass spectrometry, NMR, and vibrational spectroscopy.^31,33,34^ These isotopes can be added to growth media and are subsequently incorporated into the structure of amino acids/proteins, fatty acids, and nucleic acids through standard biochemical pathways.^35^ This method can be combined with AST approaches to assess bacterial activity and AMR at both single-cell and population levels.^13,36,37^ In this study, we aimed to develop a rapid approach for AST by integrating REIMS methodology with deuterium isotope probing (DIP), metabolomics, and multivariate statistical analysis. This novel workflow enabled detection of AMR in UPEC isolates, addressing the need for faster diagnostics to improve clinical decision-making and combat the spread of AMR.

## Experimental Methods

### Chemicals, Microorganisms, and Growth Conditions

Unless otherwise stated, all chemical compounds utilized in this research were purchased from Sigma-Aldrich (United Kingdom). Several UPEC isolates,^38^ were chosen and cultured on Luria–Bertani (LB) agar to attain axenic colonies from –80 °C glycerol stocks. Before inoculation in LB broth, each isolate underwent three subcultures on LB agar plates overnight at 37 °C. To culture the UPEC isolates in broth media, colonies were chosen from the cultured agar plate and inoculated into 50 mL of fresh LB broth in 250 mL conical flasks. The cultures were incubated at 37 °C in an Infors HT Minitron incubator shaker (Infors HT, Switzerland) at 180 rpm for 18 h.

### Quality Assurance Samples Preparation

To minimize the impact of nonexperimental factors and account for potential variation in sample analysis and instrumental performance over a diagnostically relevant time-period (several days), quality assurance (QA) samples consisting of a single biological replicate (*n* = 1) of a UPEC isolate were prepared based on the details mentioned in the growth conditions section and aliquoted into several tubes. To ensure consistency, QA samples were analyzed between every 5–6 sample during REIMS analysis.

### Optimization of D_2_O Concentration

*E. coli* MG1655 was prepared (3 biological replicates, each measured in triplicate) and incubated with defined concentrations of D_2_O, ranging from 0 to 80% at intervals of 10%, followed by the growth conditions described earlier. After the incubation period of 30 and 60 min, samples were subjected to centrifugation at 5,000 × *g* for 10 min using a standard chilled benchtop Eppendorf centrifuge 5910 R (Eppendorf Ltd., Cambridge, UK) at 4 °C. The resulting supernatant was discarded, and the biomass pellet was washed three times in 1 mL of sterile physiological saline (0.9% NaCl) solution. The washed biomass was stored at – 80 °C prior to further analysis.

### Identifying the Origin of D_2_O: Differentiation of Intracellular/extracellular D_2_O and Biotic/Abiotic Processes

After optimizing the concentration of D_2_O, *E. coli* MG1655 was cultured (3 biological replicates, each measured in triplicate) to investigate the origin of D_2_O and the potential for distinguishing between intracellular and extracellular heavy water origin. Prior to centrifugation and preparation procedures, each bacterial culture was split into two aliquots. One set of harvested biomass for each condition was subjected to sonication using a 340-W, 60 *Hz*, FisherBrand 15053 benchtop ultrasonic bath (FisherBrand, Massachusetts, USA) for 15 min. Sonication was performed to inactivate the bacteria and ensure that any observed deuterium exchange or incorporation was purely nonbiological in origin. The second set was prepared following our standard protocol (without sonication) as described previously. The samples were then stored at – 80 °C until subsequent analysis

### Rapid Trimethoprim AST for UPEC Isolates Using REIMS at the Community Level

Selected UPEC isolates (*n* = 6) were cultured (4 biological replicates) under experimental conditions in M9 minimal medium supplemented with 5 g L^–1^ casamino acids and 50 mg L^–1^ adenine (MMCAA) (Table S.1), with or without 10% D_2_O and with or without 10 mg L^–1^ trimethoprim (TMP). TMP prevents the growth of *E. coli* by inhibiting folic acid synthesis, which interferes with DNA replication and cellular division, particularly in minimal media where growth is dependent on the supplementation of amino acids and adenine. The bactericidal effects of TMP are more evident in minimal media compared to rich media like LB, which contains a broader range of nutrients and can mitigate the effects of TMP on bacterial growth.^39,40^ The growth media was therefore shifted from LB to M9 minimal medium supplemented with casamino acids and adenine to better assess TMP’s impact on the bacterial metabolism and antimicrobial resistance mechanisms. Cultures were then grown in 40 mL volumes within 50 mL Falcon tubes (FisherBrand, Fisher scientific, UK) at 37 °C and shaken at 180 rpm for 30 and 60 min. Following incubation, the cultures were centrifuged at 5,000 × *g* for 20 min using an Eppendorf 5810 G benchtop centrifuge at 4 °C (Eppendorf Ltd., Cambridge, UK). The supernatant was discarded, and the biomass pellet was washed once with 1 mL of sterile physiological saline (0.9% NaCl). The washed biomass was then stored at –80 °C for subsequent analysis.

### REIMS Sample Preparation and Analysis

Bacterial biomasses were resuspended in Milli-Q/heavy water (20 μL) and transferred onto precut 3 × 3 cm Whatman GF/C glass microfiber filter papers (Whatman plc, Kent, UK), which do not introduce any background MS signal A modified electrosurgical probe, also known as an iKnife,^41^ with infrastructural transfer tubing (Medres, Budapest, Hungary) was utilized to create aerosols containing intracellular and extracellular metabolites. This procedure was conducted within a safety biocabinet to ensure containment and operator safety. The monopolar hand-piece unit was connected to an electrosurgical generator (Erbe Medical Ltd., Germany) set to monopolar ″cut″ mode at 15 W power. The cut mode in electrosurgery uses continuous, high frequency alternating current (AC) to concentrate energy on a small area. This creates intense heat that rapidly vaporises tissues/samples, enabling precise cutting/analyzing with minimal charring or coagulation. Multiple bacterial sampling points were used to create the aerosols. The produced aerosols were channelled to a mass analyzer (SYNAPT G2-Si, Waters corp., Manchester, UK) through a 1.5 m long PTFE tube (Erbe Medical Ltd., Germany). The samples were analyzed in negative ionization mode. The power was applied by pressing the hand piece’s button, while the blade gently touched the surface of the biomass for 2–5 s. The blade was cleaned with sandpaper followed by wiping with isopropyl alcohol (propan-2-ol or IPA) after 3–5 distinct vapor acquisitions. The instrument was calibrated using sodium formate (HCOONa) solution at concentration of 5 mM as per the manufacturer’s instructions. IPA was introduced into the system as the solvent matrix, with a flow rate of 50 μL/min. An external lock mass compound of leucine enkephalin (Leu-enk), with a mass of *m*/*z* 554.2616 in negative-ion mode, was utilized in correcting mass drift for all bacterial samples. Data were collected using a scanning range of *m*/*z* 50–1200 and a scan time of one second, in resolution mode.

## Data Analysis

All collected REIMS raw data were imported to Waters Offline Model Builder software (OMB) version: 1.0.0.0 (Waters Research Centre, Budapest, Hungary). The data were then preprocessed by performing lock mass correction (negative ionization mode = *m*/*z* 554.2616), background subtraction, and mass binning to 0.1 Da. With a mass range of *m*/*z* 50 to 1200, the processed data was transformed into data matrices and saved for additional analysis. Data processing, normalization, and statistical analysis were carried out using MATLAB version 2023a (Mathworks, USA). To reduce the impact of high similarity features, sample sets underwent data filtering using interquartile range,^42^ while median normalization, square root transformation, and Pareto scaling were utilized to decrease the significance of large-fold changes within the data set. The data analysis procedure was optimized by generating different models and altering the *m*/*z* range window, with mass ranges *m*/*z* 600 to 1000. The REIMS data were then subjected to multivariate data analysis using the principal component analysis (PCA) method to identify natural clustering patterns.^43^ PCA was chosen due to its ability to reduce dimensionality while preserving the maximum variance in the data set, allowing the identification of major trends and sources of variation. Given the high-dimensional nature of mass spectrometry data, PCA was essential for extracting key features and visualizing relationships between samples in an unsupervised manner. By transforming the data into a series of new orthogonal variables (principal components), PCA provides insight into sample distribution and potential grouping patterns.^44,45^

## Results and Discussion

### Optimization of D_2_O Concentration

A total of 12 samples of *E. coli* MG1655 cultured in varying concentrations of D_2_O, ranging from 0% to 80% at 10% intervals, were collected and analyzed using REIMS. The mass spectra of *E. coli* MG1655 cultured in LB (Figure 1 **-A**) and LB containing 10% D_2_O (Figure 1 **-B**) were obtained within the range of *m*/*z* 50–1200. These spectra display signal intensities across various *m*/*z* values, including the *m*/*z* 600–900 range, which corresponds to complex lipids^46^ and is primarily dominated by intact structural phospholipids.^28^ Deuterium incorporation into the structural phospholipids of *E. coli* MG1655 was evident in the isotopic patterns of the mass spectra. In unlabeled samples cultured in LB (Figure 1 **-A**), high-intensity bins associated with phospholipids such as phosphatidic acid (PA), phosphatidylethanolamine (PE) and phosphatidylglycerol (PG) are prominent (*m*/*z* 600–900).^47,48^ In contrast, samples labeled with deuterium (Figure 1 **-B**) exhibit these bins fragmented into multiple smaller bins and shifted to the higher *m*/*z* region. This fragmentation results from the replacement of hydrogen atoms with deuterium within the lipid structures, indicating varying levels of isotopic incorporation.

**Figure 1:**
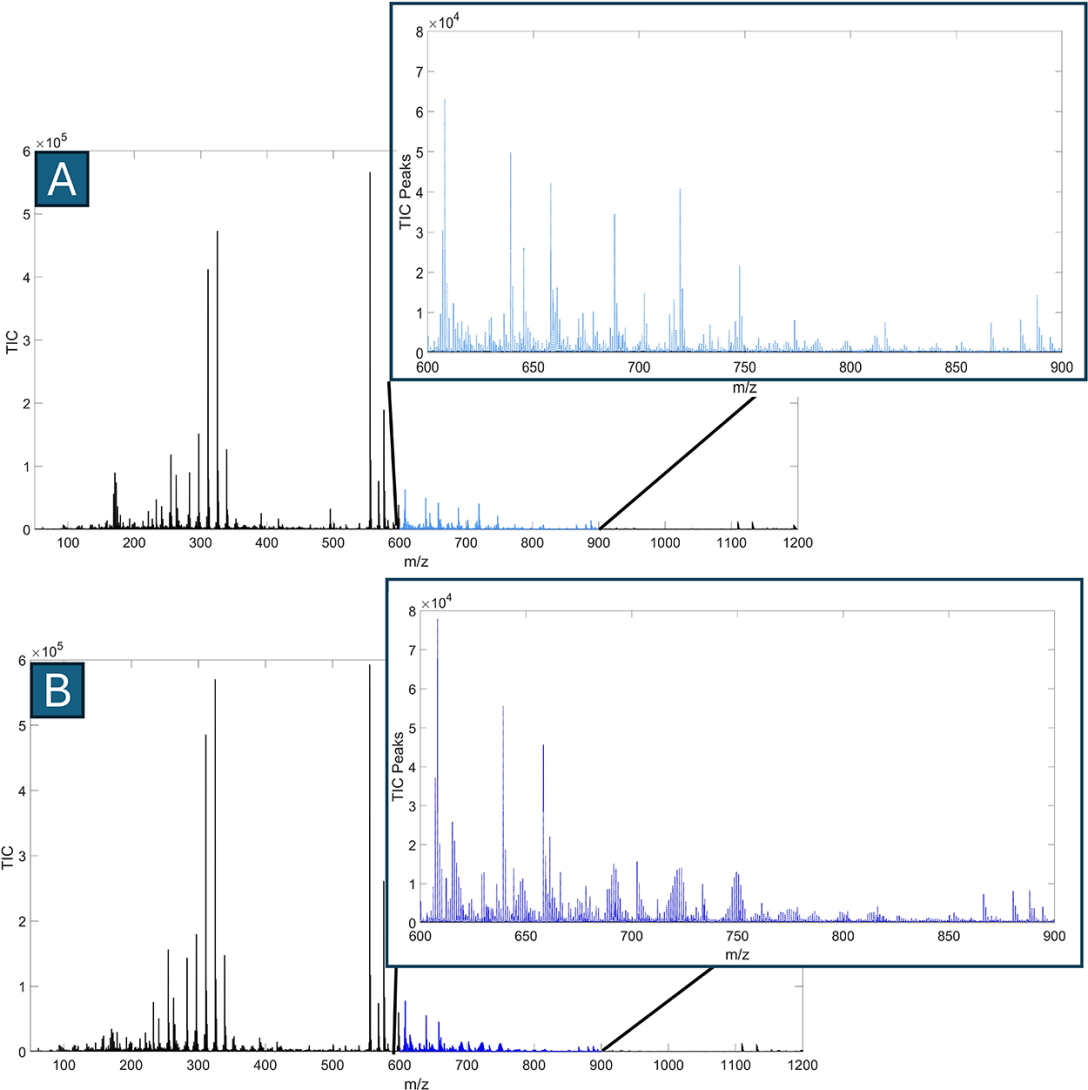
Representative mass spectra of an *E. coli* MG1655 cultured in LB (A) and LB containing 10% D_2_O (B), showing a mass range of *m*/*z* 50–1200 with an emphasis on complex lipids (*m*/*z* 600–900). Different colors indicate growth conditions: LB (light blue) and LB with 10% D_2_O (dark blue).

The PCA scores plot of REIMS data for *E. coli* MG1655 exposed to different concentrations of D_2_O for 1 h accounted for 47.63% of the total explained variance (TEV) (Figure 2A). The plot demonstrated clear discrimination between bacterial cells cultured in LB with varying D_2_O concentrations (0–80%). Notably, deuterium incorporation induced a nonlinear shift in the clustering pattern, depicted by the curved arrow in the figure, corresponding to increasing D_2_O levels. Bacterial groups cultured in D_2_O concentrations less than 40% formed distinct clusters along both PC1 and PC2 axis, indicating the major contribution of these principal components to their separation (Figure 2A). This discrimination is probably due to limitations imposed by the effects of the H/D kinetic isotope effect on bacterial growth rate and metabolism.^30,49,50^

**Figure 2:**
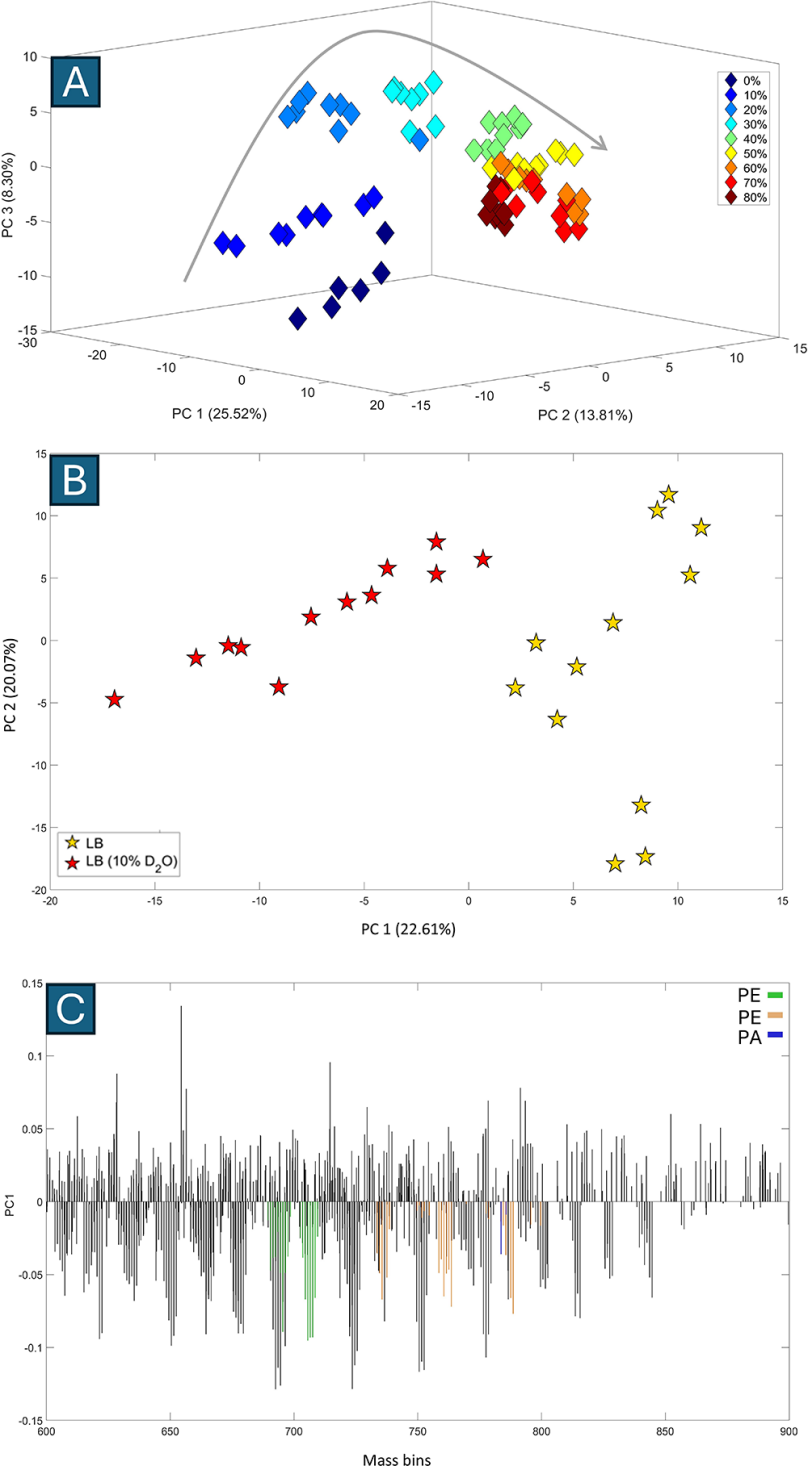
3D PCA scores plot of REIMS data for *E. coli* MG1655 at the 1 h time point (TEV = 47.63%), cultured in LB with varying concentrations of D_2_O (A). Colored diamonds indicate the different concentrations of D_2_O used in the culture medium, with numbers denoting the percentage of D_2_O. Gray arrow illustrates the trend in deuterium incorporation levels. PCA scores plot (TEV: 65.78%) of REIMS data for *E. coli* MG1655 grown in LB (yellow pentagrams) and LB containing 10% D_2_O (red pentagrams) after 1 h (B). PC1 loadings plot of REIMS data for *E. coli* MG1655 at the 1 h time point, showing bins that contributed most to the clustering patterns observed (C). Colored bins indicate binned values that fall within the expected ranges for deuterium-labeled phospholipids such as phosphatidic acid (PA; blue), phosphatidylethanolamine (PE; green), and phosphatidylglycerol (PG; orange), based on prior literature. These assignments remain tentative as no structural confirmation was performed. The legend provides a color key for these putative phospholipid groupings.

To optimize experimental and cost efficiency, and to identify the minimum concentration of D_2_O required for distinguishing bacterial groups based on deuterium incorporation, separate models were created using REIMS data from bacterial groups cultured in LB and LB containing varying concentrations of D_2_O. These data sets were then analyzed using PCA to identify clustering patterns. The PCA scores plot of *E. coli* MG1655 REIMS data at the 1-h time point (Figure 2 **-B**) revealed a clear separation between the bacterial group cultured in LB (yellow pentagrams) and the group cultured in LB with 10% D_2_O (red pentagrams) along the PC1 axis. The PC1 loadings plot (Figure 2 **-C**) highlighted the most influential bins for distinguishing these groups, with mass shifts on the negative side of PC1 associated with the group cultured in D_2_O, reflecting deuterium incorporation into the phospholipids. It has been reported that both heterotrophic and autotrophic organisms incorporate a substantial number of water-derived protons (or D^+^ ions when D_2_O is present) during the reduction of NADP and NADH. These H^+^/D^+^ ions are transferred from water (H_2_O) or D_2_O to electron carriers involved in cellular metabolism and subsequently incorporated into lipids through fatty acid biosynthesis.^51–53^ The resulting fatty acids (acyl chains) are then esterified to glycerol-3-phosphate, forming precursors like PA. These precursors are further converted into mature phospholipids such as PE and PG through the de novo biosynthesis pathway.^54,55^ Thus, theoretically, as acyl chains are synthesized independently and subsequently esterified to glycerol-3-phosphate, all hydrogen atoms can potentially be replaced by deuterium during fatty acid biosynthesis.

To aid the interpretation of significant bins that differentiated labeled from unlabeled bacterial cells in the PC1 loadings plot, theoretical *m*/*z* bin values were calculated to represent possible deuterium incorporation patterns in the acyl chain structures of commonly reported *E. coli* phospholipids, including [PE (32:1)-H]^−^, [PG (33:1)-H]^−^, [PG (36:2)-H]^−^, and [PA (42:2)-H]^−^. Several bins on the negative side of PC1 showed mass shifts that may correspond to these predicted values; however, given the use of 0.1 Da binning and the absence of structural confirmation, these associations remain uncertain. The bins with theoretical matches are highlighted in different colors in Figure 2-C for interpretive reference.

To investigate whether specific bins reflect deuterium incorporation into phospholipid structures, we analyzed the average mass spectra (*n* = 96) obtained from UPEC isolates, including both TMP-sensitive and TMP-resistant strains, cultured in MMCAA supplemented with 10% D_2_O. Spectra were normalized against three reference bins at *m*/*z* 688.45, 733.55, and 773.45. These *m*/*z* values have been previously reported in the literature and attributed to PE(32:1), PG(33:1), and PG(36:2) species, respectively, based on high-resolution accurate mass and MS/MS fragmentation data.^21^ While this prior study supported their putative identity, no MS/MS analysis was performed in the present study, and therefore, the bin assignments reported here remain tentative.

Bar chart plots of the D/H ratios over time revealed a clear increasing trend (Figures S1A–L and S2A–L), consistent with time-dependent deuterium uptake. This observation supports the notion that these bins likely capture metabolic incorporation of deuterium into lipophilic components of the cell, although precise compound identification cannot be confirmed within the current experimental framework. The high standard deviation observed in some groups likely reflects technical variability introduced during manual REIMS analysis, including differences in biomass loading and ionization efficiency.

It is also important to note that the use of a relatively large binning window (0.1 Da), in combination with high levels of deuterium incorporation, contributes to spectral complexity by increasing the likelihood of multiple ion species being grouped into a single bin. This bin-level overlap reduces annotation confidence and complicates interpretation of compound identity. However, this complexity does not impact the ability of REIMS combined with D_2_O labeling to sensitively detect AMR, as the AMR-related classification relies on global spectral differences and multivariate pattern recognition rather than specific compound assignments. Importantly, the ability to detect AMR-related metabolic differences using combination of REIMS with D_2_O does not depend on precise identification of individual mass spectral bins. Rather, the approach relies on global spectral patterns and time-dependent deuterium uptake trends, which reflect metabolic activity and are sufficient for discriminating resistant and susceptible isolates.

Furthermore, it is worth mentioning that a total of 96 spectra were generated from 24 biological replicates (six UPEC isolates, each with four independent cultures), with four technical replicates acquired per biological replicate. Biological replicates were used as the basis for statistical interpretation and multivariate analysis (e.g., PCA), while technical replicates were averaged for quantification purposes such as D/H ratio calculations. This design preserves biological variability while reducing the risk of overfitting due to technical replication and ensures the robustness of the conclusions drawn. Although each technical replicate was treated as an individual data point in the PCA models, averaging them per biological replicate produced comparable clustering patterns, indicating that the observed separation between susceptible and resistant isolates is driven by true biological variation rather than technical artifacts.

Next, to explore the distinction between intracellular and extracellular deuterium incorporation, as well as the biological versus chemical nature of deuterium incorporation, PCA was performed on REIMS data from *E. coli* MG1655 under eight distinct conditions. The data indicated that deuterium incorporation primarily occurs through biological processes rather than extracellular exchange. Further details and analyses are provided in the Supporting Information (Table S2 Figures S3A-C and S4).

To evaluate the application of REIMS and DIP for identifying AMR in UPEC isolates, all REIMS data were preprocessed and analyzed using clustering techniques after incubation under conditions outlined in the methods section. The 3D PCA scores plot of all samples, accounted for 42.16% of TEV­(Figure S5), revealed clear discrimination between samples grown in H_2_O and D_2_O, regardless of TMP treatment. Specifically, both treated and untreated groups of UPEC isolates grown in MMCAA supplemented with 10% D_2_O, at both 30 min and 60 min time points, clustered together. In contrast, samples grown in H_2_O (0, 30, and 60 min time points), as well as the 0 min time point of samples grown in MMCAA supplemented with 10% D_2_O, clustered together with all QA samples (comprising reference UPEC isolate) since no detectable deuterium incorporation occurred. The tight clustering of QA samples (Figure S5) demonstrated high reproducibility and consistent instrument performance, despite the absence of batch-to-batch correction. To better illustrate the effects of growth and TMP treatment in the presence or absence of D_2_O, PCA scores plots of REIMS data for UPEC isolates were analyzed separately, based on D_2_O and H_2_O conditions (Figure 3). In the PCA scores plot for samples grown in D_2_O (Figure 3 **-A**), combined effects of growth, deuterium incorporation, and antibiotic treatment, led to a time-dependent clustering pattern along the PC1 axis, with 0 min samples clustering on the negative side and 60 min samples on the far positive side. While no clear separation between resistant (diamonds) and susceptible (circles) isolates was observed at 0 min, by 30 min, TMP treatment, combined with deuterium incorporation, caused the treated susceptible isolates (empty circles with dark green outlines) to begin separating from the untreated susceptible group (dark green circles) due to reduced deuterium uptake. This trend persisted at 60 min, where treated susceptible isolates (empty circles with dark red outlines) remained closer to the 30 min samples due to lower deuterium incorporation, whereas resistant isolates (both treated and untreated) and untreated susceptible isolates continued shifting further along the positive side of PC1. For samples grown in H_2_O (Figure 3 **-B**), a slight trend was observed but was not apparent. However, separation occurred along the PC2 axis, primarily reflecting the effects of growth and TMP treatment. Additionally, the 30 and 60 min samples clustered closely together, indicating weaker discrimination due to growth effects. While a subtle separation between treated susceptible isolates and other groups was observed at 30 and 60 min, it was less evident than in D_2_O conditions, emphasizing the role of deuterium in enhancing discrimination.

**Figure 3:**
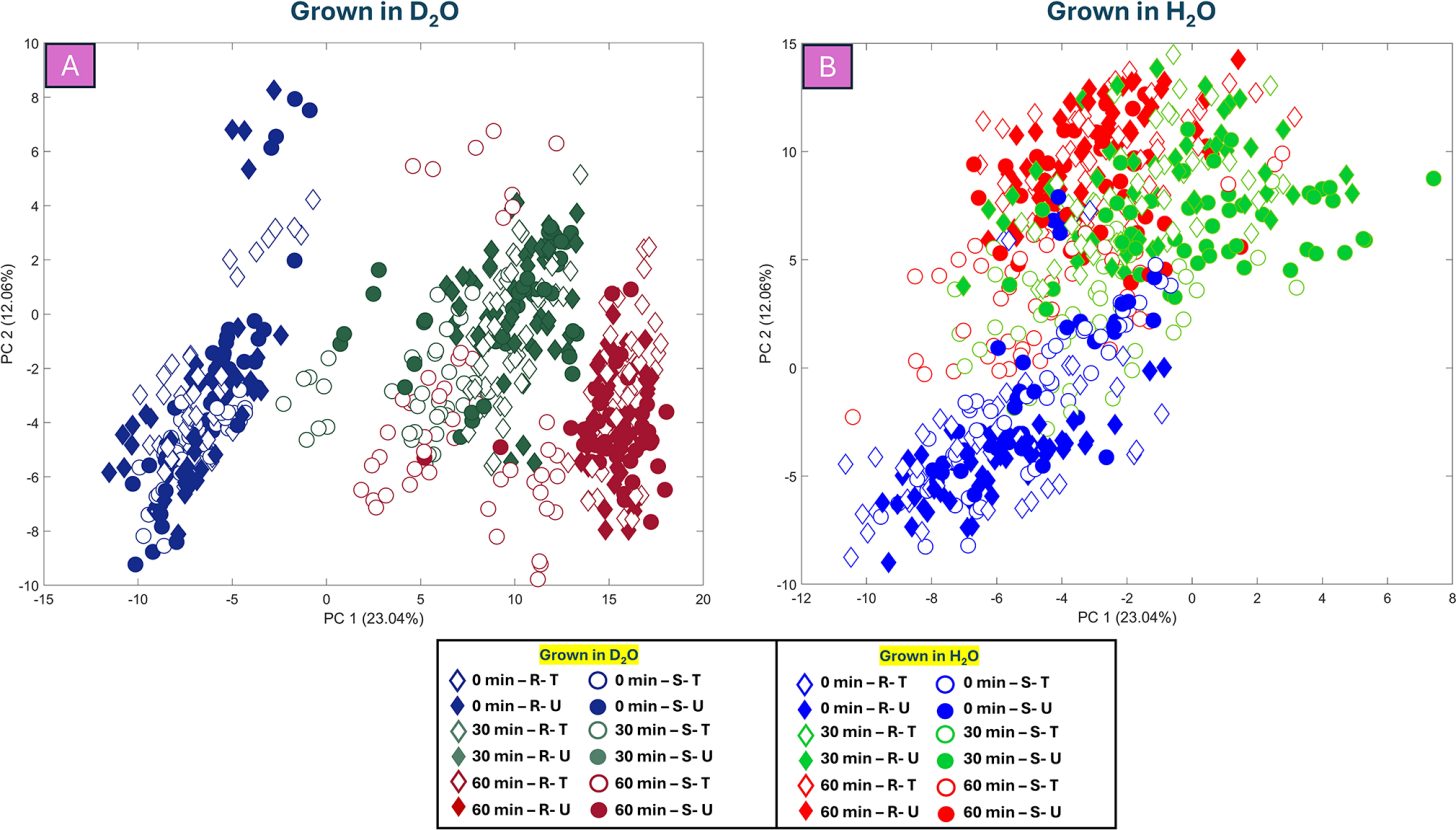
PCA scores plot (TEV: 35.10%) displays REIMS data for UPEC isolates grown in MMCAA with (A) or without (B) 10% D_2_O and with (T) or without (U) TMP treatment. Colors represent sampling time points: Blue for 0 min, green for 30 min, and red for 60 min. Experimental conditions are distinguished by color shading: darker shades indicate growth in D_2_O, while lighter shades indicate growth in H_2_O. TMP-treated samples are marked as empty symbols. Diamonds correspond to TMP-resistant isolates (R), and circles to TMP-susceptible isolates (S). The legend provides a color key to interpret the figure.

To enhance the visualization of the discriminating effects of TMP combined with deuterium, the PCA score plots were simplified by removing certain sample groups and displaying them separately for TMP-sensitive and TMP-resistant UPEC isolates grown in MMCAA with or without D_2_O (Figure 4). For TMP-resistant isolates grown in D_2_O (Figure 4 **-A**; TEV: 35.10%), a time-dependent pattern was observed along the PC1 axis, driven by growth and deuterium incorporation. However, no clear discrimination was evident between TMP-treated and untreated groups, indicating similar levels of deuterium uptake as expected. In contrast, the PCA scores plot for TMP-susceptible isolates grown in D_2_O (Figure 4 **-B**; TEV: 35.10%) showed a similar time-dependent pattern along PC1, but with subtle separation at 30 min due to TMP-induced growth inhibition and reduced deuterium incorporation. This separation more evident at 60 min, where treated TMP-susceptible isolates clustered separately from untreated groups, reflecting their diminished ability to incorporate deuterium due to TMP treatment. For TMP-resistant isolates grown in H_2_O (Figure 4 **-C;** TEV: 35.10%), a time-dependent trend was observed along the PC2 axis, with 0 min samples clustering on the negative side and both 30 and 60 min samples grouping together on the positive side. However, no clear separation was observed between TMP-treated and untreated groups, suggesting no significant effect of TMP on resistant isolates. Furthermore, additional analysis focusing on the 60 min time point confirmed that this separation was consistently observed across all biological replicates and was not driven by any single biological replicate or sample (data not shown). Similarly, in TMP-susceptible isolates grown in H_2_O (PCA scores in Figure 4 **-D**; TEV: 35.10%), a time-dependent trend was noted, with minimal discrimination between 0, 30, and 60 min samples. While some separation between treated and untreated groups was observed along the PC2 axis at 30 min and became more distinct at 60 min, the effect remained weaker compared to D_2_O conditions. This highlights the role of deuterium in enhancing TMP-induced discrimination and underscores its importance for rapid AST of UPEC isolates using REIMS within 1 h. Optical density (OD) measurements at 600 nm taken during the experiment (Figure S6 A-F) revealed no significant differences in growth rates between treated and untreated groups of TMP-susceptible and TMP-resistant UPEC isolates. This suggests that bacterial responses to antibiotics may not be detectable through OD measurements within such a short time frame. Furthermore, variations in culturing conditions, heterogeneity among isolates, differing levels of sensitivity to TMP, and potential pipetting errors, make it challenging to rely on OD readings alone for accurate AMR detection in such short incubation times.^56,57^ Given these limitations, advanced approaches such as REIMS are essential for achieving rapid and reliable AMR identification.

**Figure 4:**
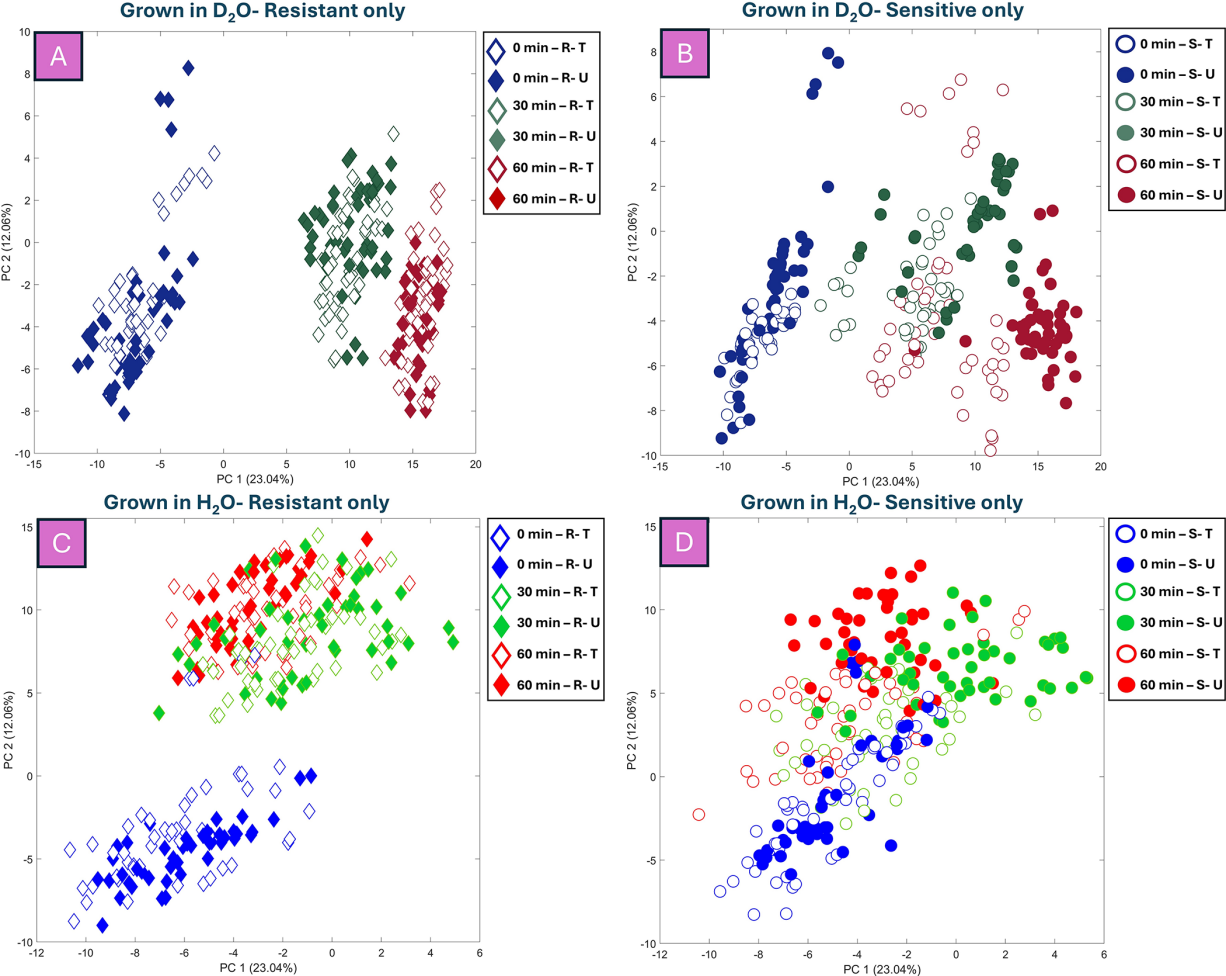
PCA scores plot of REISM data of UPEC isolates (TEV: 35.10%) grown in MMCAA with (A and B) or without (C and D) 10% D_2_O and with (T) or without (U) TMP. Colors represent sampling time points: Blue for 0 min, green for 30 min, and red for 60 min. Experimental conditions are distinguished by color shading: darker shades indicate growth in D_2_O (A and B), while lighter shades indicate growth in H_2_O (C and D). TMP-treated samples are marked as empty symbols. Diamonds correspond to TMP-resistant isolates (R), and circles to TMP-susceptible isolates (S). The legend provides a color key to interpret the figure.

## Conclusions

In this study, and to the best of our knowledge, we report for the first time the application of REIMS coupled with DIP and multivariate statistical analysis for rapid AST of UPEC isolates within a 1-h incubation period. The use of D_2_O proves particularly powerful, since water is central to numerous metabolic pathways, including fatty acid biosynthesis, meaning that deuterium is rapidly incorporated into cellular structural components. Our results also demonstrated consistent and reproducible clustering patterns in the REIMS mass spectra, highlighting the robustness of this technique when employed following optimized protocols. The incorporation of deuterium into bacterial phospholipids produced distinct isotopic signature patterns, and multivariate analysis revealed significant clustering based on deuterium incorporation. Clear differentiation was observed for TMP-treated and untreated susceptible isolates grown in MMCAA with 10% D_2_O for 60 min, while no such differentiation was seen in the control condition lacking D_2_O. However, further optimization is needed to improve the sensitivity and speed of AMR detection for clinical use. In conclusion, this study highlights the potential application of REIMS, combined with DIP, as an innovative and efficient fingerprinting tool for rapid evaluation of AMR in a very short time-period. Future studies could explore automating the sample analysis platform and establishing protocols to account or control variations in the biomass quantity analyzed during each REIMS analysis. Moreover, the findings reported in this initial study could be extended by using infrared laser-assisted REIMS for more accurate, controlled sample analysis, and improved reproducibility. Additionally, future investigations may explore the transferability of this approach to different bacterial strains and evaluate its applicability with different antibiotics, broadening its potential utility in antimicrobial susceptibility testing

## Supplementary Material



## Notes

Additional experimental details, including culture conditions and REIMS analysis parameters; composition of M9 minimal medium (Table S1); overview of experimental conditions for deuterium incorporation study (Table S2); average REIMS mass spectra for TMP-sensitive and TMP-resistant UPEC isolates (Figures S1–S2); PCA score plots and supporting interpretation of REIMS data (Figures S3–S5); bar charts of OD_600_ measurements showing TMP susceptibility profiles (Figure S6) (PDF)
