# Supplementary material for: Application of Rapid Evaporative Ionization Mass Spectrometry (REIMS) to Identify Antimicrobial Resistance in Uropathogenic Escherichia coli (UPEC) Isolates via Deuterium Isotope Probing
Source: Anal Chem. 2025 Aug 22;97(34):18444–52. doi: 10.1021/acs.analchem.5c00667 (PMC12409694; doi:10.1021/acs.analchem.5c00667)
Supplement: Supplementary file 1 [file ac5c00667_si_001.pdf]

Supporting Information

Application of Rapid Evaporative Ionisation Mass Spectrometry (REIMS) to Identify Antimicrobial Resistance in Uropathogenic *Escherichia coli* (UPEC) Isolates *via* Deuterium Isotope Probing

Sahand Shams[1], Sara Sadia Chowdhury[1], Joel Doherty[1], Shwan Ahmed[1], Dakshat Trivedi[1], Yun Xu[1], Joscelyn Sarsby[2], Claire Eyers[2], Adam Burke[1], Royston Goodacre[1], Howbeer Muhamadali[*1]

[1]Centre for Metabolomics Research, Department of Biochemistry, Cell and Systems Biology, Institute of Systems, Molecular and Integrative Biology, University of Liverpool, Liverpool, L69 7ZB, United Kingdom

[2] Centre for Proteome Research, Department of Biochemistry, Cell and Systems Biology, Institute of Systems, Molecular and Integrative Biology, University of Liverpool, Liverpool, L69 7ZB, United Kingdom

Correspondence author email address: Howbeer.Muhamad-Ali@liverpool.ac.uk

---

Table of Content

- Table S1: Composition of M9 minimal medium used for the growth of *E. coli* K-12 MG1655 (Page S2)
- Table S2: Distinct experimental conditions to assess D_2O incorporation (Page S2)
- Figure S1: Average REIMS spectra of UPEC isolates (*m/z* 688.45) (Page S3)
- Figure S2: Average REIMS spectra of UPEC isolates (*m/z* 733.55 and 773.45) (Page S4)
- Figure S3: PCA plots for *E. coli* MG1655 under various D_2O/H_2O conditions (Page S5)
- Figure S4: PCA plot showing order of sample analysis (Page S5)
- Figure S5: 3D-PCA plots for UPEC isolates with/without TMP (Page S6)
- Figure S6: OD600 bar charts of UPEC isolate cultures (Page S7)

To explore the possibility of distinguishing between intracellular and extracellular D_2O, as well as investigating the biological or chemical nature of deuterium incorporation, a database comprising eight distinct conditions was generated (Table S2). Here, chemical deuterium incorporation refers to H/D exchange at labile hydrogen positions, while biological incorporation involves the metabolic integration of deuterium into cellular structures. These conditions were determined based on the culture medium used (LB or LB supplemented with 10% D_2O), whether the sample was sonicated or not, and whether the analysis was conducted using pure H_2O or D_2O. Subsequently, PCA was employed to detect any clustering patterns present within the dataset generated under the above conditions. The PCA scores plot of the REIMS data of *E. coli* MG1655 (**Figure S3-A**), accounted for 39.13% of TEV, displayed a significant degree of discrimination along the PC 1 axis between the D_2O-grown bacterial group and the bacterial cells grown in H_2O. This is while no discrimination was observed between the groups analysed using H_2O (circles), and D_2O (diamonds), indicating that extracellular D_2O does not have a significant effect on the REIMS signal (**Figure S3-A**). Furthermore, it was observed that the H_2O-grown sonicated bacterial group analysed with D_2O (indicated by dark blue circles with black outlines) and the D_2O-grown sonicated group analysed with H_2O (indicated by bright diamonds with black outlines) were clearly discriminated and clustered separately suggesting that deuterium incorporation is a result of a biological process. To investigate the spread of the data/drift of the data through the PCA scores plot of the REIMS data of *E. coli* MG1655 (**Figure S4**) and the operation of the instrument, another set of PCA scores was plotted where the data points were labelled according to the order of samples analysis, to examine any potential systematic instrument drifts that might have contributed to the spread observed in the PCA scores plot (**Figure S4**). The results showed that the spread observed previously was not correlated with the order of sample analysis. Furthermore, to enhance the exploration of the biological or chemical nature of deuterium incorporation, the REIMS data were divided into two sets: one comprising data from LB-grown cells and the other containing data from cells grown in LB media supplemented with 10% D_2O. These datasets were subsequently analysed using PCA to identify any clustering patterns present within them. The PCA scores plot of the REIMS data of cells grown in LB containing 10% D_2O (**Figure S3-B**), accounted for 41.24% of TEV, did not show any clear discrimination between the ones analysed with H_2O and D_2O, indicating that the extracellular D_2O does not affect the signals obtained from the bacteria grown in the presence of heavy water, meaning that the deuterium incorporation is a biological procedure. The PCA scores plot of REIMS data of the cells grown in LB (**Figure S3-C**), accounted for TEV: 37.52% of TEV, indicated a subtle separation between the sample groups

that were analysed with H_2O and those analysed using D_2O based on PC2 axis, suggesting that a chemical deuterium incorporation is taking place wherein deuterium atoms are replacing hydrogen atoms during the ionisation This is further supported by the observation that both sonicated and non-sonicated cells analysed with D_2O cluster closely together, forming a mixed cluster. This confirmed that deuterium incorporation after harvesting the cells was not due to bacterial metabolic activity, as even the inactive bacterial cells (under sonicated conditions) cluster together. It is noteworthy that the variation between the H_2O and D_2O groups was only evident in bacterial samples grown in LB, and not in those grown in LB with 10% D_2O. This is likely due to the greater impact of biological processes as opposed to chemical processes.

Table S1: Composition of M9 minimal medium used for the growth of *E. coli* K-12 MG1655

Chemical Compounds	Chemical Formula	Concentration (gL^{-1})
D-Glucose	$C_6H_{12}O_6$	5
Ammonium chloride	NH_4Cl	1
Magnesium sulfate heptahydrate	$MgSO_4.7H_2O$	0.5
Sodium hydrogen phosphate	Na_2HPO_4	3
Potassium dihydrogen phosphate	KH_2PO_4	3
Ferrous sulfate heptahydrate	$FeSO_4.7H_2O$	0.001
Calcium chloride	$CaCl_2$	0.001

TableS2. Distinct conditions investigated to differentiate intracellular and extracellular D_2O and explore nature of deuterium incorporation mechanism.

**Culture media**	**Sonication Status**	**REIMS Analysis**
LB	Sonicated	Analysed with D_2O
		Analysed with H_2O
	Not Sonicated	Analysed with D_2O
		Analysed with H_2O
LB containing 10% D_2O	Sonicated	Analysed with D_2O
		Analysed with H_2O
	Not Sonicated	Analysed with D_2O
		Analysed with H_2O

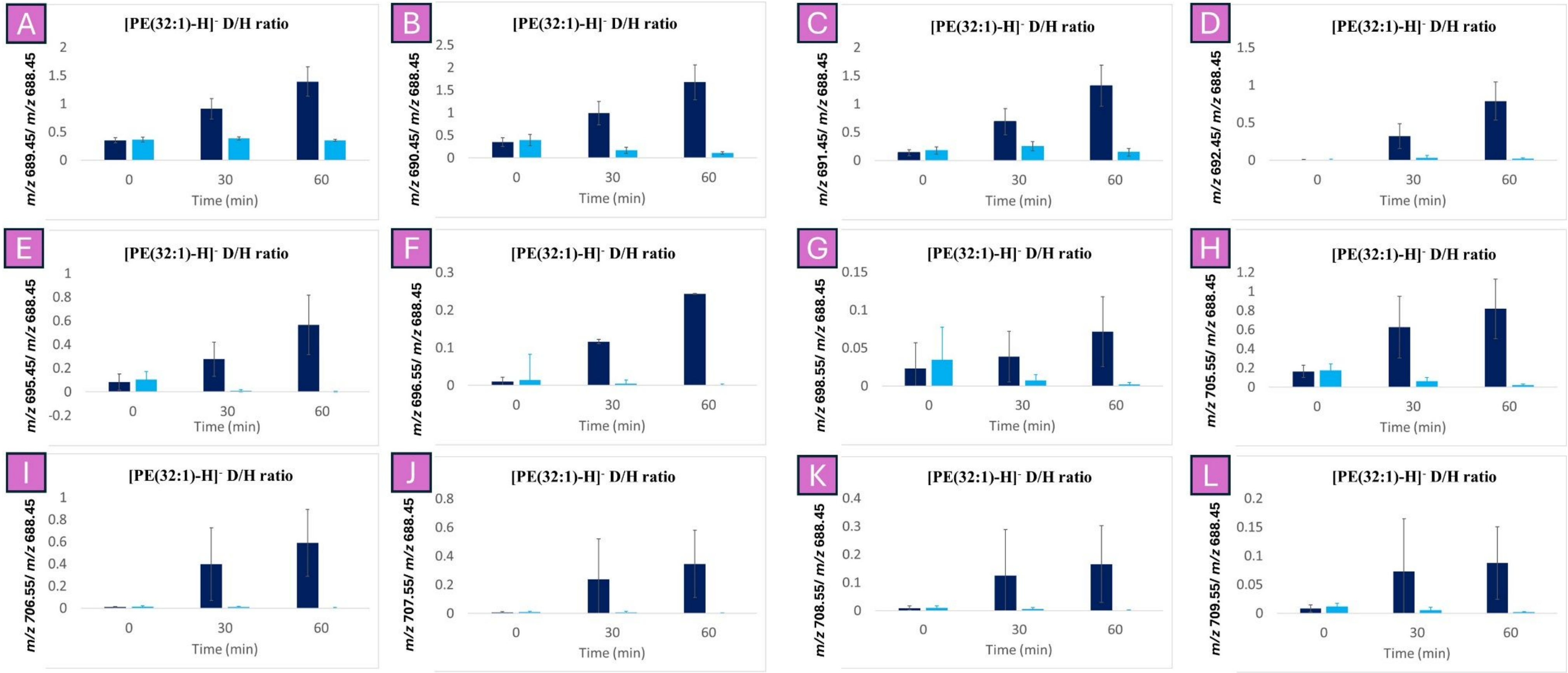

Figure S1. Average mass spectra (n = 96) of selected bins from the REIMS data of experimental UPEC isolates, including both TMP-sensitive and TMP-resistant strains, cultured in MMCAA supplemented with 10% D_2O. Spectra were derived from 24 biological replicates (6 isolates × 4 independent cultures), with 4 technical replicates per biological replicate. Data were normalised against an unlabelled bin at m/z 688.45, which falls within the expected range for PE species (A–L). Different colours represent various growth conditions. Error bars indicate ± standard deviation.

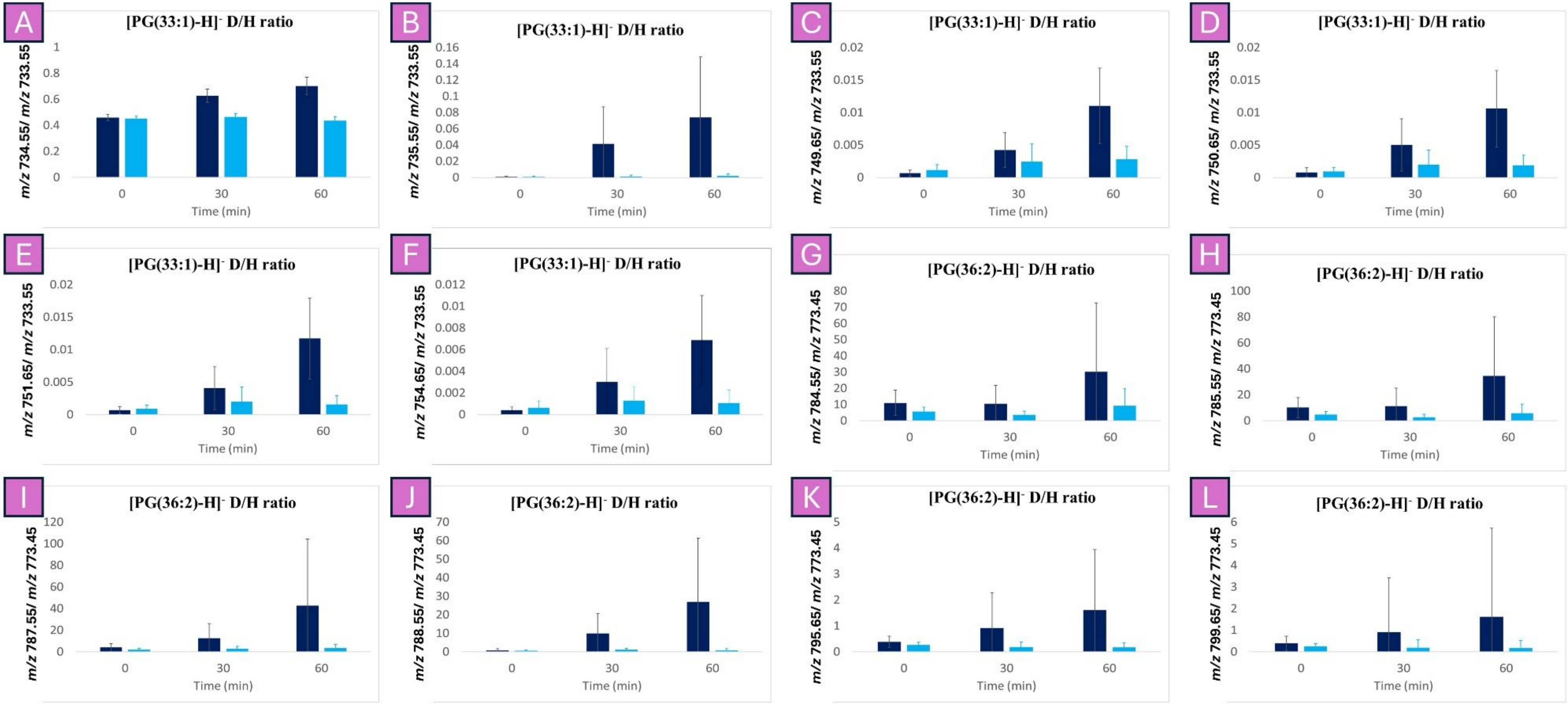

Figure S2. Average mass spectra (n = 96) of selected bins from the REIMS data of experimental UPEC isolates, including both TMP-sensitive and TMP-resistant strains, cultured in MMCAA supplemented with 10% D_2O. Spectra represent 24 biological replicates (6 isolates × 4 independent cultures), with 4 technical replicates per biological replicate. Data were normalised against unlabelled bins at m/z 733.55 and 773.45, which fall within the expected range for PG (33:1) (A–F) and PG (36:2) (G–L), respectively, based on literature reports. Different colours represent various growth conditions. Error bars indicate ± standard deviation.

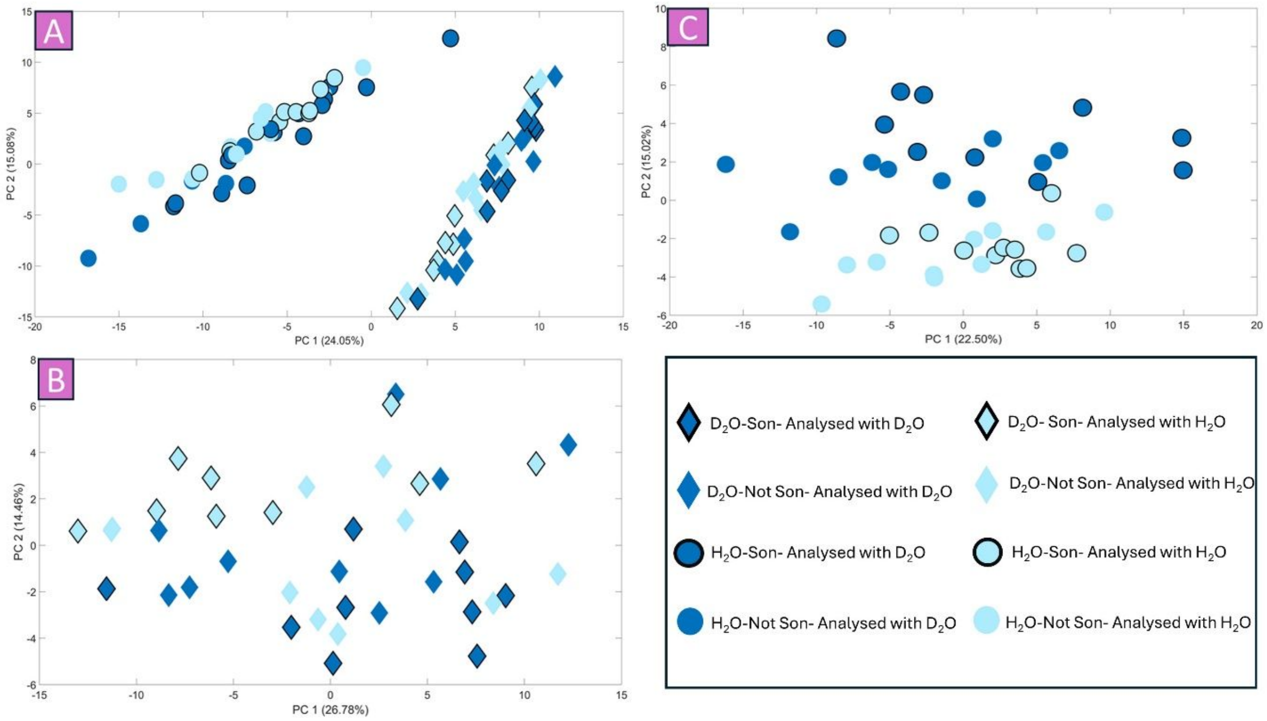

Figure S3-A: PCA scores plot of REIMS data (*m/z* 600-900) of *E. coli* MG-1655 at 1 h time (TEV:39.13%) point grown in LB media with (diamonds) or without (circles) D_2O. Different colours represent different types of water including light (bright blue) and heavy water (dark blue), used in this experiment. The black outline highlights the sonicated (Son) condition, while the not sonicated (Not Son) condition is represented by the absence of the black outline. **B and C:** PCA scores plot of REIMS data of *E. coli* MG1655 at 1 h time point grown in LB containing 10% D_2O, represented as diamonds (TEV:41.24%) **(B)** and LB represented as circles (TEV: 37.52%) (**C**). Different colours represent different types of water including light (bright blue) and heavy water (dark blue), used in this experiment. The black outline highlights the sonicated (Son) condition, while the not sonicated (Not Son) condition is represented by the absence of the black

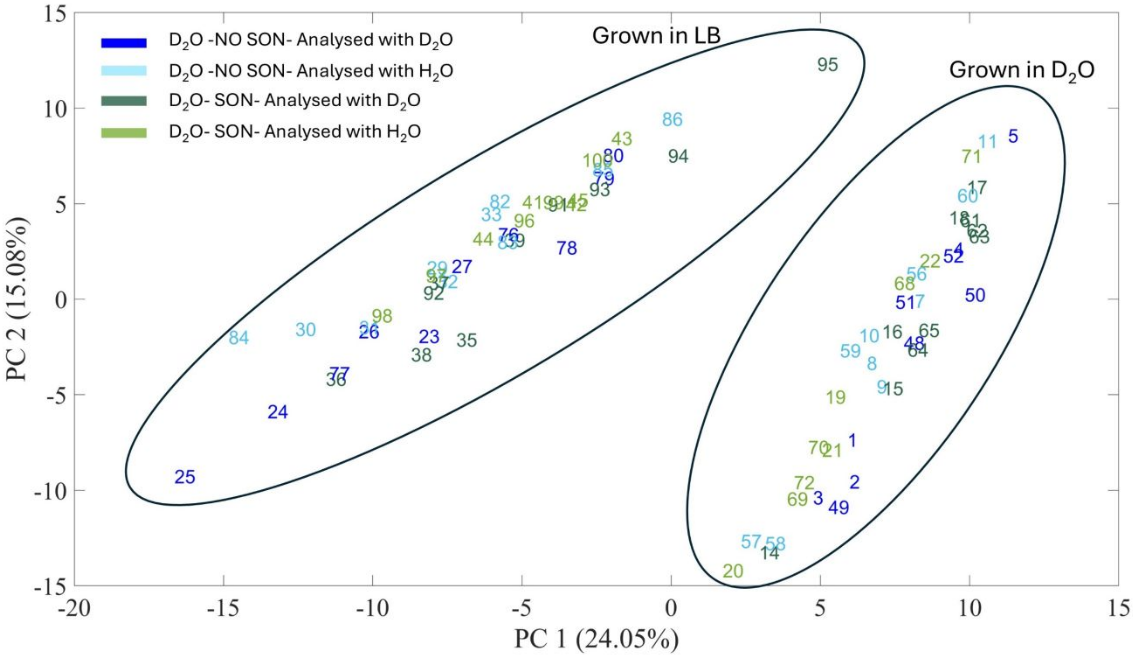

Figure S4. PCA scores plot of REIMS data (600–900 *m/z*) for E. coli MG1655 at the 1-h time point. Samples were grown in LB media with or without D_2O, sonicated (green shades) or non-sonicated (blue shades), and analysed using D_2O (darker shades) or H_2O (lighter shades). Data points are labelled based on the order of analysis. The legend provides a colour key for interpreting the figure.

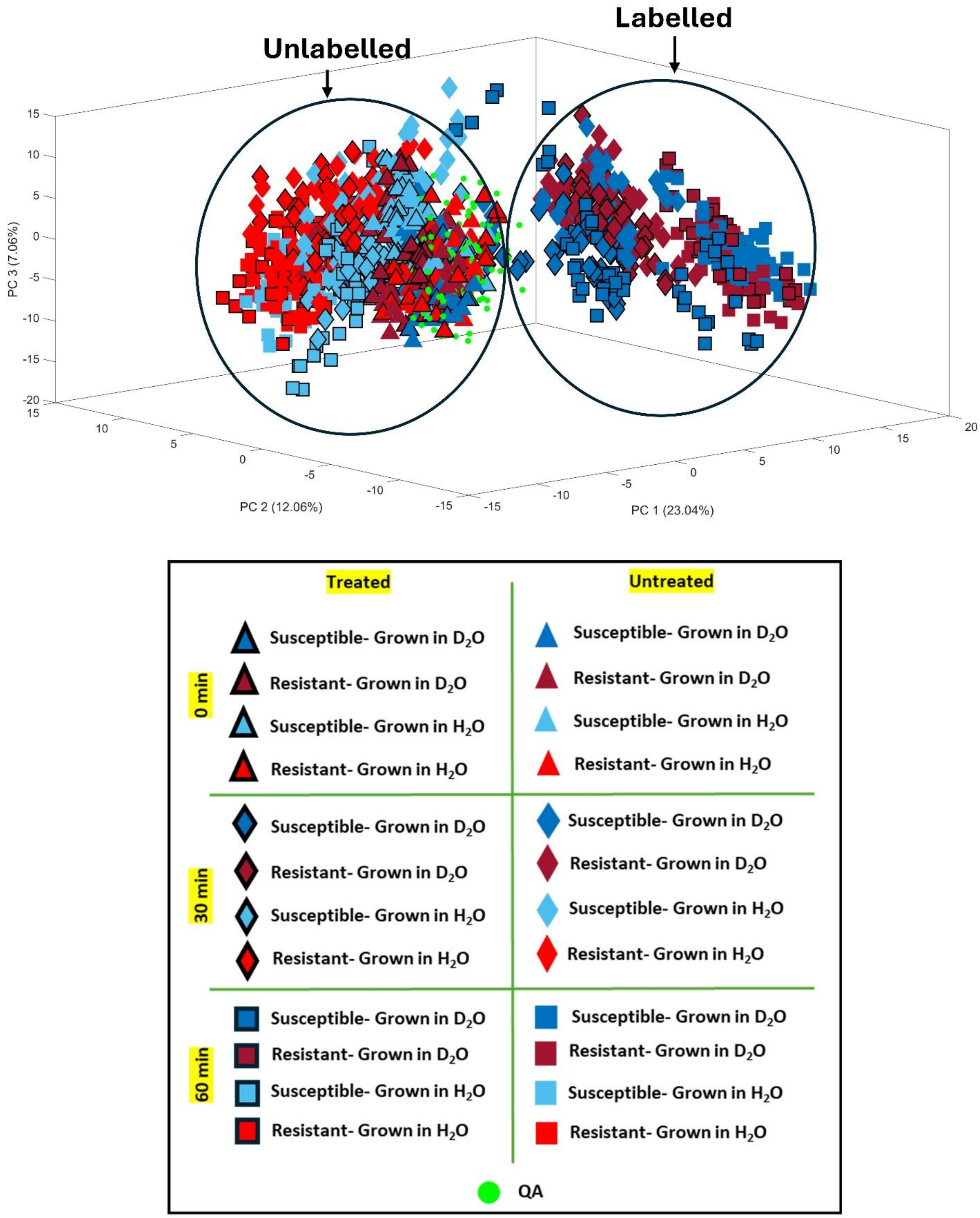

Figure S5. 3D-PCA scores plot visualises REIMS data for UPEC isolates grown in MMCAA with or without 10% D_2O and with or without TMP. Symbols represent sampling time points: triangles for 0 min, diamonds for 30 min, and squares for 60 min. Experimental conditions are distinguished by colour shading: darker shades indicate growth in D_2O, while lighter shades indicate growth in H_2O. TMP-treated samples are marked with a black outline. Red tones correspond to TMP-resistant isolates, and blue tones to TMP-susceptible isolates. QA refers to the single bacterial isolate that was used throughout the experiment to test for reproducibility in the REIMS analysis. The black circles highlight the labelled and unlabelled groups. The legend provides a colour key to interpret the figure.

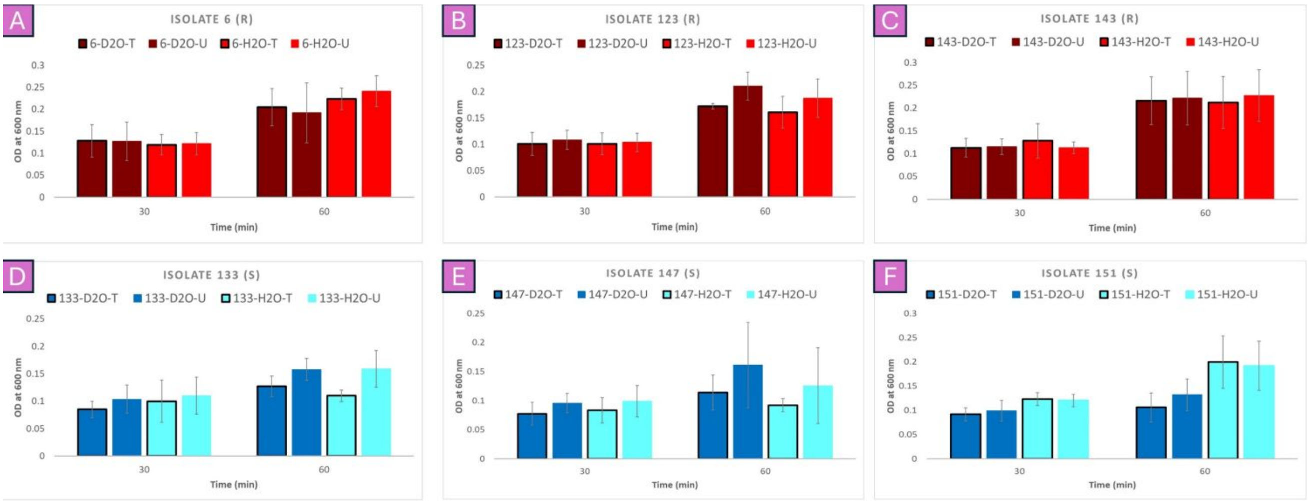

Figure s6. Bar charts showing the average of OD measurements (n=4) at 600 nm of UPEC isolate cultures at 30 and 60 min. Bars are colour-coded to indicate TMP susceptibility: red for TMP-resistant (R) isolates **(A-C)**, and blue for TMP-sensitive (S) isolates **(D-F).** Error bars indicate ± standard deviation.
